# Studies on the Thermal Decomposition Course of Nitrogen-Rich Heterocyclic Esters as Potential Drug Candidates and Evaluation of Their Thermal Stability and Properties

**DOI:** 10.3390/ijms25094768

**Published:** 2024-04-27

**Authors:** Marta Worzakowska, Krzysztof Sztanke, Małgorzata Sztanke

**Affiliations:** 1 Department of Polymer Chemistry, Institute of Chemical Sciences, Faculty of Chemistry, Maria Curie-Sklodowska University in Lublin, 33 Gliniana Street, 20-614 Lublin, Poland; marta.worzakowska@mail.umcs.pl; 2 Laboratory of Bioorganic Compounds Synthesis and Analysis, Medical University of Lublin, 4A Chodźki Street, 20-093 Lublin, Poland; krzysztof.sztanke@umlub.pl; 3 Department of Medical Chemistry, Medical University of Lublin, 4A Chodźki Street, 20-093 Lublin, Poland

**Keywords:** triazinylformic acid ethyl esters, triazinylacetic acid methyl esters, decomposition course, thermal behaviour, thermal degradation products, toxicity to erythrocytes assessment, antihaemolytic activity

## Abstract

Drug candidates must undergo thermal evaluation as early as possible in the preclinical phase of drug development because undesirable changes in their structure and physicochemical properties may result in decreased pharmacological activity or enhanced toxicity. Hence, the detailed evaluation of nitrogen-rich heterocyclic esters as potential drug candidates, i.e., imidazolidinoannelated triazinylformic acid ethyl esters **1**–**3** (where R_1_ = 4–CH_3_ or 4–OCH_3_ or 4–Cl, and R_2_ = –COOC_2_H_5_) and imidazolidinoannelated triazinylacetic acid methyl esters **4**–**6** (where R_1_ = 4–CH_3_ or 4–OCH_3_ or 4–Cl, and R_2_ = –CH_2_COOCH_3_)—in terms of their melting points, melting enthalpy values, thermal stabilities, pyrolysis, and oxidative decomposition course—has been carried out, using the simultaneous thermal analysis methods (TG/DTG/DSC) coupled with spectroscopic techniques (FTIR and QMS). It was found that the melting process (documented as one sharp peak related to the solid–liquid phase transition) of the investigated esters proceeded without their thermal decomposition. It was confirmed that the melting points of the tested compounds increased in relation to R_1_ and R_2_ as follows: **2** (R_1_ = 4–OCH_3_; R_2_ = –COOC_2_H_5_) < **6** (R_1_ = 4–Cl; R_2_ = –CH_2_COOCH_3_) < **5** (R_1_ = 4–OCH_3_; R_2_ = –CH_2_COOCH_3_) < **3** (R_1_ = 4–Cl; R_2_ = –COOC_2_H_5_) < **1** (R_1_ = 4–CH_3_; R_2_ = –COOC_2_H_5_) < **4** (R_1_ = 4–CH_3_; R_2_ = –CH_2_COOCH_3_). All polynitrogenated heterocyclic esters proved to be thermally stable up to 250 °C in inert and oxidising conditions, although **1**–**3** were characterised by higher thermal stability compared to **4**–**6**. The results confirmed that both the pyrolysis and the oxidative decomposition of heterocyclic ethyl formates/methyl acetates with *para*-substitutions at the phenyl moiety proceed according to the radical mechanism. In inert conditions, the pyrolysis process of the studied molecules occurred with the homolytic breaking of the C–C, C–N, and C–O bonds. This led to the emission of alcohol (ethanol in the case of **1**–**3** or methanol in the case of **4**–**6**), NH_3_, HCN, HNCO, aldehydes, CO_2_, CH_4_, HCl, aromatics, and H_2_O. In turn, in the presence of air, cleavage of the C–C, C–N, and C–O bonds connected with some oxidation and combustion processes took place. This led to the emission of the corresponding alcohol depending on the analysed class of heterocyclic esters, NH_3_, HCN, HNCO, aldehydes, N_2_, NO/NO_2_, CO, CO_2_, HCl, aromatics, and H_2_O. Additionally, after some biological tests, it was proven that all nitrogen-rich heterocyclic esters—as potential drug candidates—are safe for erythrocytes, and some of them are able to protect red blood cells from oxidative stress-induced damage.

## 1. Introduction

**Figure 1:**
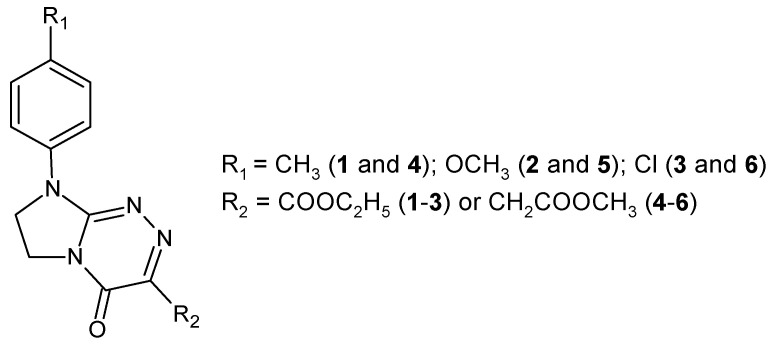
Structures of ethyl 4-oxo-8-[4-(R-phenyl)]-4,6,7,8-tetrahydroimidazo[2,1-*c*][1,2,4]triazine-3-carboxylates (**1**–**3**) and methyl 2-{4-oxo-8-[4-(R-phenyl)]-4,6,7,8-tetrahydroimidazo[2,1-*c*][1,2,4]triazin-3-yl}acetates (**4**–**6**), which have been thermally evaluated in this article.

Imidazolidinoannelated triazinylformic acid ethyl esters (**1**–**3**) and imidazolidinoannelated triazinylacetic acid methyl esters (**4**–**6**) (Figure 1) belong to two important classes of molecules with the prospective antitumour or antinociceptive field of relevance, respectively, whose structures in solution and solid state have been established [1,2,3,4,5,6]. They were designed as functionalised isosteric isomers of azacytosine. Having the amino nitrogen locked up in an imidazolidine ring, these innovative small molecules may be of biochemical interest as they are suspected to be resistant to enzymatic deamination, unlike short-acting cytosine- or azacytosine-based drugs (such as cytarabine, gemcitabine, or azacytidine, respectively), which are susceptible to inactivation by cytidine deaminase [7,8,9]. Therefore, their development may contribute to the discovery of more effective and metabolically stable azaisocytosine-based drugs for anticancer chemotherapy. To enter the individual tumour cells or the central nervous system, their structures had to be hydrophobic enough to cross the biological membranes, including the blood–brain barrier. In this case, the presence of a certain number of non-polar moieties and a temporarily masked polar group were required. The possibility of using two types of nitrogen-rich heterocyclic esters (**1**–**3** and **4**–**6**) as potential future drugs has previously been extensively studied [1,2,3,4,5,6]. It was found that these two types of compounds reveal a low toxicity in vitro and in vivo [1,2,3,5]. Among heterocyclic ethyl formates, the structure **2**—bearing a *para*-methoxy-substituted phenyl moiety—has been disclosed as the most promising anticancer agent candidate. In the 5-bromo-2′-deoxyuridine-based assay assessing DNA synthesis and cell proliferation, this compound was low in toxicity for normal HSF (human skin fibroblast) cells, evoked an antiproliferative effect in human breast carcinoma (T47D) and cervical carcinoma (HeLa) cells higher than that of a clinically used anticancer drug pemetrexed, and was also potent at inhibiting the growth of both thalidomide-resistant and thalidomide-susceptible human multiple myeloma cells (MM1R and MM1S, respectively). The flow cytometric analysis showed that the mode of its anticancer action was connected with the cell cycle arrest (at the S and G_2_/M phases) in human cervical (HeLa), ovarian (TOV112D—human ovarian primary malignant adenocarcinoma cells) and breast (T47D) tumour cells. Moreover, by observing the stained cells under a confocal microscope, it was confirmed that the death of cervical carcinoma cells induced by this compound is necrosis-dependent [3]. In addition, the molecule **2** was used to design a non-selective adenosine A_2A_ receptor antagonist, which was then patented as a drug [10]. The first analytical method for the quantitative determination of the potential drug candidate **2** in human serum samples was recently developed as well [6]. Among heterocyclic methyl acetates, the structure **4**—containing a *para*-methyl-substituted phenyl moiety—proved to be the most promising analgesic agent candidate. This small molecule with relatively low toxicity in mice showed significant in vivo antinociceptive activity after intraperitoneally induced hyperalgesia [5]. Reliable QSAR models and the statistically significant correlations between lipophilicity indices and pharmacokinetic descriptors—which have been developed in our previous chromatographic studies—predict that both groups of heterocyclic esters (**1**–**3** and **4**–**6**) have good bioavailability and high permeability through biological barriers, including cell membranes [4,5]. Because of favourable biopharmaceutical properties, they may be promising anticancer agent candidates or potential analgesics deserving further research.

On the other hand, a number of molecules having the protected acid function as an ester are prodrugs used to increase permeability through biological membranes and consequently bioavailability. The following are only a few examples of ester prodrugs used in medicine: oseltamivir (the ethyl ester prodrug of an antiviral oseltamivir acid), tenofovir disoproxil fumarate (a prodrug of tenofovir with antiviral activity), pivampicillin, talampicillin, bacampicillin (prodrugs being acyloxymethyl esters of an antibiotic ampicillin), sultamicillin (a hybrid ester prodrug of an ampicillin—aminopenicillin and sulbactam—a β-lactamase inhibitor), candoxatril (the indanyl ester prodrug of a protease inhibitor candoxatrilate) or enalapril (the ethyl ester prodrug of an antihypertensive agent enalaprilate). These more lipophilic molecules, due to the attachment of less polar ester moiety, easily cross cell membranes and are finally activated via hydrolysis in the blood by esterases, releasing the parent drugs with free acid function [11,12,13].

Thermal analysis methods, such as differential scanning calorimetry (DSC) and thermogravimetry/differential thermogravimetry (TG/DTG), are helpful in the design and development of potential drugs. They allow for the testing of a whole range of biomolecules, both organic and inorganic. The DSC method is commonly used to study the stability of molecules, the course of melting, denaturation, gelling, evaporation, crystallisation, polymorphic transformations, and heat of transformation. The TG/DTG technique primarily enables the investigation of the behaviour of compounds during heating, the identification of degradation products, as well as the understanding the thermal decomposition mechanism [14].

The literature survey revealed a limited number of studies on the thermal behaviour of 1,2,4-triazine derivatives containing the ester functionality. Thermal stability and volatile decomposition products emitted during the pyrolysis of methyl (3,4-disubstituted-5-oxo-4,5-dihydro-1,2,4-triazin-6(1*H*)ylidene)acetates were disclosed [15]. High thermal stability has been demonstrated for the designed ethyl 7-chloro- or 7-methyl-1-(4-nitrophenyl)-1,4-dihydrobenzo[*e*][1,2,4]triazine-3-carboxylates [16]. Thermal characterisation and mesomorphic behaviour of a hybrid 1,2,4-triazine molecule containing an azomethine bridge, which was obtained by condensing 1,2,4-triazine-3-amine with 4-(3-cholesteryloxycarbonyl)benzaldehyde, was reported [17]. However, there is still a research gap in these studies as the mechanism of thermal degradation and detailed thermal properties of nitrogen-rich heterocyclic esters with the *para*-R_1_-substitution at the phenyl moiety and the ethoxycarbonyl or methoxycarbonylmethylene moiety at R_2_ (**1**–**3** and **4**–**6**, respectively) (Figure 1) are unknown. Therefore, the detailed thermal characterisation of these potential drug candidates is needed in the preclinical phase of drug development. To establish the appropriate conditions during storage, processing, and thermal utilisation, it is necessary to know about their thermal behaviour.

The purpose of the present paper is to evaluate, for the first time, both classes of heterocyclic esters (i.e., imidazolidinoannelated triazinylformates (**1**–**3**) and imidazolidinoannelated triazinylacetates (**4**–**6**)) in terms of their thermal stability, melting points and enthalpy values, and their eventual polymorphic transformations. The subsequent aim is to determine their thermal decomposition course during pyrolysis carried out under inert and oxidative conditions on the basis of the identified volatile degradants. Understanding the thermal decomposition profiles of the investigated compounds will be of high practical utility when establishing the appropriate storage, processing, and utilisation conditions. For the current comparative thermal studies, we have chosen derivatives from both classes of nitrogen-rich heterocyclic esters (i.e., ethyl formates **1**–**3** and methyl acetates **4**–**6**) that have the same *para*-substitution at the phenyl moiety denoted as R_1_ and diverse functional moieties marked as R_2_ (Figure 1). In our studies, we have applied the simultaneous TG/DTG/DSC coupled with spectroscopic methods (i.e., FTIR and QMS), because these reliable techniques are preferred in the thermal analyses of medicines and therapeutic agent candidates in the preclinical phase of drug development. They enable the determination of thermal stability, heat of fusion, the identification of volatile degradation products, as well as checking the purity (including a content of water and solvents used in the crystallisation process) and possible solid–solid transitions [14,15,18,19,20,21,22,23]. This entire research is aimed at understanding the beneficial thermal properties of these compounds, which may increase their usefulness as potential candidates for pharmaceutical applications.

## 2. Results and Discussion

### 2.1. The Melting Points and the Melting Enthalpies Evaluated by the DSC

**Figure 2:**
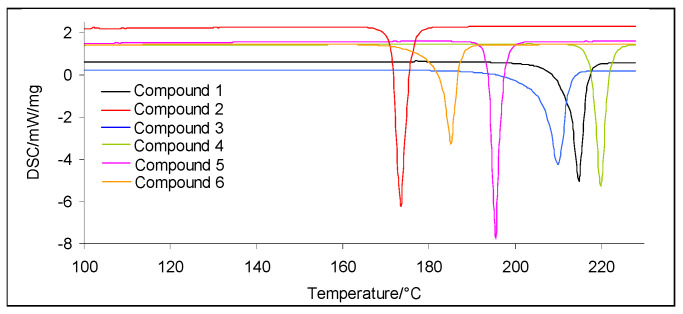
The DSC curves for the tested compounds (**1**–**6**) collected in a synthetic air atmosphere.

Figure 2 shows the DSC curves for the tested compounds collected in an inert atmosphere. The values of melting points (*T*_onset_ and *T*_melt_) and the melting enthalpies (Δ*H*) determined in helium and air atmospheres are presented in Table 1.

The solid–liquid phase transition of each tested nitrogen-rich heterocyclic ester is visible as one sharp endothermic peak (Figure 2), confirming that each analysed molecule has been synthesised as a pure compound. This is as expected since high purity of the investigated compounds has previously been confirmed based on spectral, HPLC, and electrochemical results [1,2,4,5,6]. The obtained results indicate that the studied esters have the melting point above 170 °C in both atmospheres, and they do not decompose because no mass loss is observed during their melting. In addition, the melting point values for the investigated compounds do not depend on the type of experimental atmosphere and are similar in inert and oxidising conditions. In the case of ethyl formates **1**–**3** with the *para*-R_1_-substitution at the phenyl moiety, the highest melting point is observed for compound **1** containing –CH_3_. This molecule begins to melt at a temperature of 212–213 °C, with the maximum melting temperature at 215 °C. In turn, compound **2** containing –OCH_3_ has the lowest onset and maximum melting point (170–171 °C and 172–173 °C, respectively). Summing up, the onset and maximum melting points for ethyl formates **1**–**3** depend on R_1_ and increase in the following order: **2** (R_1_ = –OCH_3_) < **3** (R_1_ = –Cl) < **1** (R_1_ = –CH_3_). However, the DSC results show that for methyl acetates **4**–**6** with the *para*-R_1_-substitution at the phenyl ring, the melting points depend on the molecular mass. As the molar mass increases, a decrease in the melting point is observed. Among these heterocyclic esters, compound **4** containing –CH_3_ shows the highest melting point (*T*_melt_ 217–221 °C). However, compound **6** with –Cl is characterised by the lowest melting point (*T*_melt_ 182–185 °C). To sum up, the onset and maximum melting points for methyl acetates **4**–**6** depend on R_1_ and increase in the following order: **6** (R_1_ = –Cl) < **5** (R_1_ = –OCH_3_) < **4** (R_1_ = –CH_3_). As it is well visible, all the *para*-R_1_-substituted esters have slightly lower values of melting enthalpies (Δ*H*) in an inert atmosphere compared to Δ*H* values in an oxidative atmosphere. The difference in Δ*H* values between furnace atmospheres is from 8.3 J/g to 11.5 J/g. In addition, in both atmospheres, the highest Δ*H* value among ethyl formates **1**–**3** is observed for compound **2** containing –OCH_3_. A similar situation is in the case of methyl acetates **4**–**6**, among which compound **5** containing –OCH_3_ has the highest Δ*H* value, Table 1. While comparing both series of esters (**1**–**3** with **4**–**6**), it turns out that ethyl formates **1**–**3** show slightly higher Δ*H* values (the difference is from 8.4 J/g to 11.5 J/g). This means that slightly more energy must be supplied in the form of heat to melt 1 g of a substance [24].

Furthermore, the analysed heterocyclic esters **1**–**6** did not undergo any polymorphic transformations when screened for polymorphic behaviour at a low heating rate. This is their advantage because it is known that such crystalline compounds should be characterised by higher stability than amorphous pharmaceutical substances that are prone to polymorphic transformations [23]. The most likely reason for this is that during the recrystallisation process from organic solvents, they were able to crystallise into the most thermodynamically stable solid phase. The beneficial thermal behaviour of the investigated compounds makes them suitable for future pharmaceutical applications. Screening for polymorphism is very important, and therefore should be performed in the preclinical phase of drug development. It is known that polymorphs may differ in their physicochemical properties (i.e., melting point, solubility, and dissolution rate) which in turn may affect their pharmacological activity, pharmacodynamics, and stability. To design an appropriate and acceptable solid form of an active pharmaceutical substance, it is necessary to perform polymorphism screening in accordance with FDA recommendations [25,26]. Therefore, in the case of our nitrogen-rich heterocyclic esters **1**–**6** with prospective medical utility, this study was carried out in the early phase of drug development.

### 2.2. The TG/DTG Analyses (Inert Conditions)

**Figure 3:**
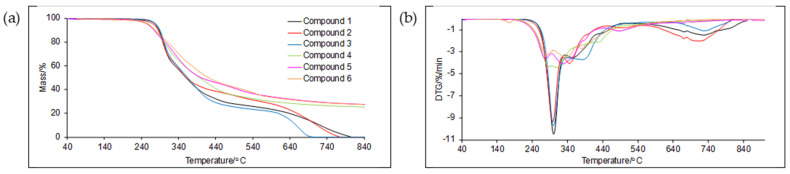
TG (**a**) and DTG (**b**) curves for the tested compounds (**1**–**6**) (inert conditions).

Figure 3 presents the TG/DTG curves of the tested nitrogen-rich heterocyclic esters for their analyses made in an inert atmosphere. Additionally, the results obtained from TG/DTG analyses are placed in Table 2. These data confirm that the *para*-substituted compounds studied are characterised by high thermal stability. Their decomposition in an inert atmosphere starts above 270 °C in the case of ethyl formates **1**–**3**, and above 250 °C in the case of methyl acetates **4**–**6**. This clearly shows that compounds **1**–**3** are characterised by approximately 13–23 °C higher thermal stability as compared to molecules **4**–**6**.

If one looks at the course of the TG/DTG curves, one can see that the thermal decomposition of the tested *para*-R_1_-substituted heterocyclic esters **1**–**6** occurs in two basic stages. The first decomposition stage between *T*_5%_ and 540 °C is clearly visible. This stage is composed of at least two/three thermal steps for ethyl formates **1**–**3**. However, for methyl acetates **4**–**6**, at least three/four thermal steps can be highlighted. This course of the TG/DTG curves indicates that the major bond breaking reactions occur one after another and/or at the same time in this temperature range. Thus, it means that the bonds contained in the structure of *para*-R_1_-substituted derivatives of the ester molecules are characterised by similar or slightly different thermal decomposition energy.

The first decomposition step between *T*_5%_ and 334 °C with *T*_max1_ at 298–301 °C and with the comparable mass loss (Δ*m*_1_*)* in the range of 36.9–40.0% for compounds **1**–**3** is observed. This temperature range and the comparable values of mass loss for *para*-R_1_-substituted ethyl formates **1**–**3** mainly indicate the breaking of one type of the bonds present in their structure. Above the temperature of 334 °C, the pyrolysis of subsequent bonds in the structure of tested compounds begins. This pyrolysis step is visible on the TG/DTG curves as another poorly resolved DTG signal with lower intensity compared to the previous one. This step spreads from 334 °C to 514 °C with *T*_max1a_ and the mass loss (Δ*m*_1*a*_*)* in the range of 31.8–37.1%. Finally, heating the compounds **1**–**3** above 514 °C results in the appearance of the second decomposition stage, with *T*_max2_ at 715–733 °C and with the mass loss (Δ*m*_2_) from 24.2% to 31.3%. These compounds decompose completely when heated to 900 °C.

However, the decomposition course of methyl acetates **4**–**6** is characterised by a very poor separation of individual steps in the first pyrolysis stage. This indicates very small differences in the energy of thermal decomposition of the individual bonds in their structure. Despite this, weakly marked maxima can be distinguished on the DTG curves, as shown in Table 2. This decomposition stage spreads from *T*_5%_ to 560 °C with the mass loss (Δ*m*_1_*_ + _*Δ*m*_1*a*_) in the range of 63.1–68.4%. Heating the compounds **4**–**6** above 560 °C does not result in the occurrence of the second DTG peak, and only a slight slow mass loss up to 900 °C is observed. The mass loss in the second decomposition stage is from 4.0% to 9.3%. These heterocyclic esters do not decompose completely when heated to 900 °C. The residual mass is comparable for particular compounds and is from 25.3% to 27.6%. This confirms the formation of derivatives with higher molecular masses during their pyrolysis. Such derivatives require higher temperatures to evaporate or decompose.

Comparing the results from TG/DTG analyses obtained for ethyl formates **1**–**3** and methyl acetates **4**–**6**, it is concluded that the decomposition of these two groups of molecules, despite their similar structure, may occur in a slightly different way, particularly after heating them to a temperature above 560 °C.

### 2.3. The Decomposition Course of the Tested Compounds in Inert Conditions

**Figure 4:**
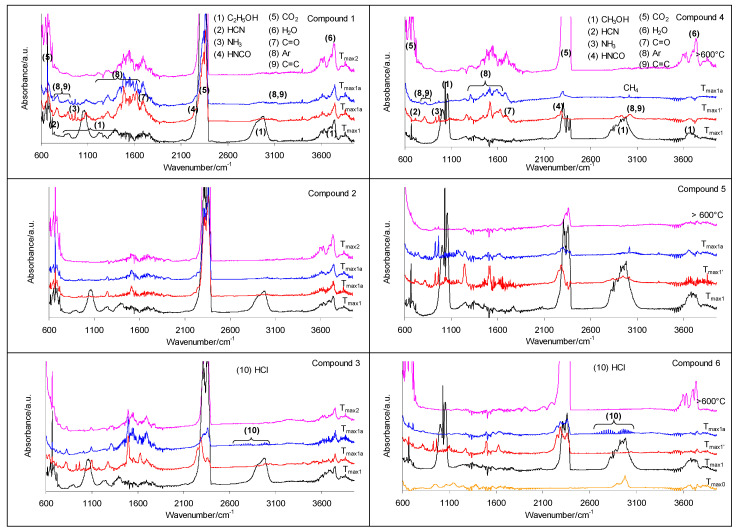
The gaseous FTIR spectra for compounds **1**–**6** collected in an inert atmosphere.

Figure 4 presents the gaseous FTIR spectra collected at the maximum characteristic temperatures obtained as a result of the heating of the tested *para*-substituted at the phenyl moiety ethyl formates **1**–**3** or methyl acetates **4**–**6**. According to the results obtained from the FTIR analysis, it can be concluded that the first decomposition step (*T*_max1_) is mainly related to the emission of alcohol, i.e., ethanol for ethyl formates **1**–**3** or methanol for methyl acetates **4**–**6**. This means that this stage is related to the pyrolysis of the ester bonds present in the structure of the tested compounds. Thus, it confirms that the ester bonds are characterised by the lowest energy of thermal decomposition among all bonds present in the structure of the analysed compounds. The formation of an alcohol molecule is confirmed by the presence of characteristic absorption vibrations for the functional groups present in the structure of heterocycles studied. In the case of ethyl formates **1**–**3**, the stretching vibrations for the OH (above 3600 cm^−1^), the stretching vibrations for the C–H (above 2780 cm^−1^ with the maximum at 2964 cm^−1^), the deformation vibrations for the C–H (1342–1390 cm^−1^), and the stretching vibrations for the C–O (1045–1230 cm^−1^) prove the formation of ethanol [27]. Meanwhile, the stretching vibrations for the OH (above 3600 cm^−1^), the stretching vibrations for the C–H (2809–2954 cm^−1^), the deformation vibrations for the C–H (1330–1345 cm^−1^), and the stretching vibrations for the C–O (998–1054 cm^−1^) indicate the emission of methanol [28] as a result of decomposition process of methyl acetates **4**–**6**.

**Figure 5:**
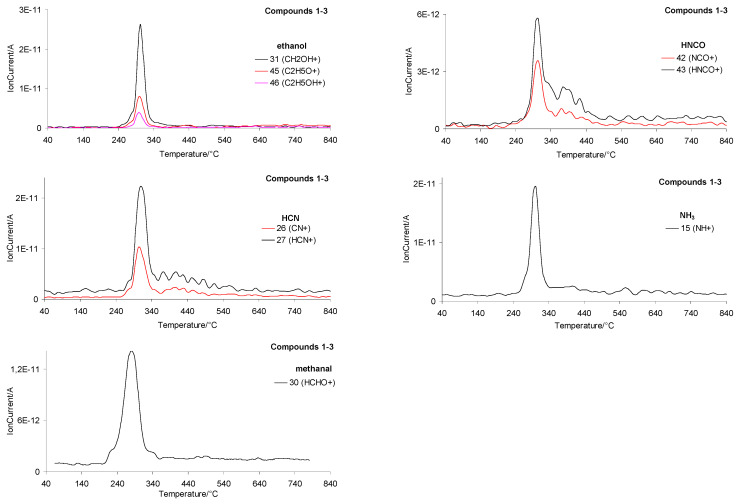

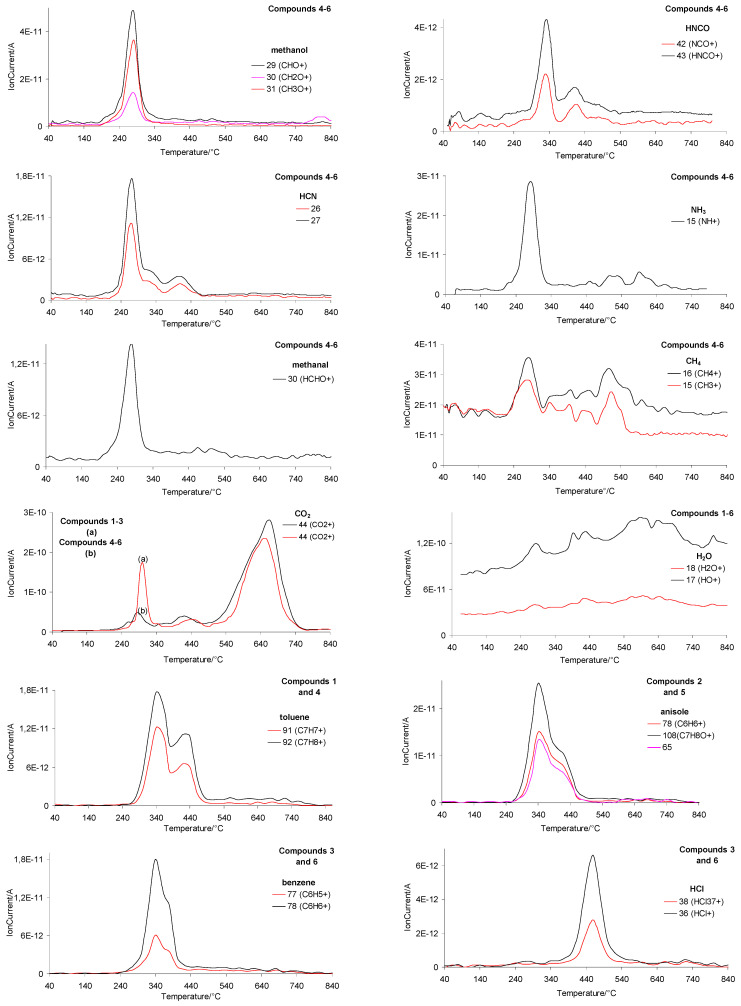
The QMS spectra for compounds **1**–**6** collected in an inert atmosphere.

Additionally, the QMS results clearly confirm the formation of suitable alcohol as a result of the cleavage of the ester bonds in the structure of the tested compounds, as shown in Figure 5. The *m/z* ions characteristic of ethanol (compounds **1**–**3**) and methanol (compounds **4**–**6**) are well visible from the QMS spectra. The ionization of ethanol leads to the formation of the *m/z* ions 31 (CH_2_OH^+^), 45 (C_2_H_5_O^+^), and 46 (C_2_H_5_OH^+^) [29], as shown in Figure 5, while the appearance of the *m/z* ions 29 (CHO^+^), 30 (CH_2_O^+^), 31 (CH_3_O^+^), and 32 (CH_3_OH^+^) confirms the emission of methanol [30] under the heating of compounds **4**–**6**. The FTIR spectra clearly indicate that alcohol with the appropriate structure is the main decomposition product of the tested compounds at *T*_max1_. At this temperature, the presence of CO_2_ as a gaseous decomposition product is also well visible. However, looking at the QMS spectra, it can be seen that in addition to alcohol, other gaseous decomposition products also begin to evolve at *T*_max1_. This indicates that the absorption bands of alcohol and CO_2_ cover the absorption bands of other volatiles due to their high intensity. Up to the temperature *T*_max1a_ for compounds **1**–**3** and *T*_max1′_ for compounds **4**–**6**, the absorption bands for the other gaseous products are clearly visible in the FTIR spectra, i.e., when the emission of alcohol is no longer observed. However, the additional QMS test confirms, which is not visible in the FTIR spectra, that a larger variety of gaseous decomposition products is already emitted from the temperature *T*_max1_. This indicates that the energies of thermal decomposition of other bonds present in the structure of the tested compounds are not much different from the energy of thermal decomposition of the ester bonds. Together with the emission of alcohol, the formation of NH_3_, HNCO, HCN, methanal, and ethene with the maximum intensity at *T*_max1a_ for compounds **1**–**3** and *T*_max1′_ for compounds **4**–**6** is observed. The formation of NH_3_ is confirmed by the presence of two absorption bands, one at 931 cm^−1^ and the other at 966 cm^−1^, which are characteristic of the deformation vibrations of N–H groups [31,32,33,34] and the attendance of *m/z* ion 15, due to the creation of NH^+^ species after the ionisation of NH_3_, as shown in Figure 4 and Figure 5. As the results indicate, the emission of ammonia is visible up to a temperature of approximately 440 °C. Also at this temperature range, the formation of HCN is confirmed by the absorption band at 713 cm^−1^ and the *m/z* ions 26 (CN^+^) and 27 (HCN^+^) [35,36]. As it is well seen from the QMS spectra, its emission up to approximately 500 °C is observed. However, the presence of HNCO in the mixture of gaseous products is confirmed by the absorption bands at 2270–2290 cm^−1^ [37,38,39] and the *m/z* ions 42 (NCO^+^) and 43 (HNCO^+^). Similarly to the emission of HCN, the release of HNCO extends to a temperature of about 500 °C. At this maximum temperature, the creation of some aldehyde compounds by the presence of the absorption bands at 1680 cm^−1^ and low intensity bands at 1720–1771 cm^−1^ responsible for the stretching vibrations for the C=O is suspected. The absorption bands at 1720–1771 cm^−1^ are characteristic of the C=O stretching vibrations of gaseous methanal. The creation of this gas is also confirmed based on presence of the *m/z* ion 30 in the QMS spectra. In addition to the emission of this aldehyde, the formation of other aldehyde-type compounds, which are formed as a result of radical reactions between different decomposition fragments is also observed (the absorption band at 1680 cm^−1^). Moreover, in the case of compounds containing –Cl at the phenyl moiety (compounds **3** and **6**), the formation of HCl is clearly seen from the gaseous FTIR spectra. Its emission by the presence of the absorption bands in the range of 2100–3100 cm^−1^ as characteristic “noises” is confirmed [40,41]. The emission of HCl for compounds **3** and **6** spreads from 400 °C to 550 °C with the maximum intensity at 480 °C.

**Scheme 1:**
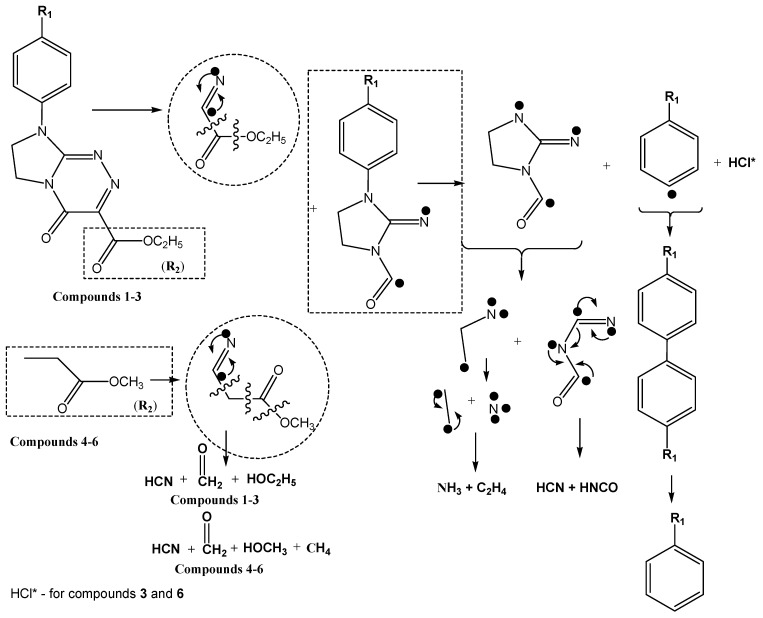
A simplified course of pyrolysis of the tested compounds (**1**–**6**) in an inert atmosphere.

In addition, the creation of CH_4_ (the band of a low intensity at 3014 cm^−1^) [41,42] for compounds **4**–**6** with the –CH_2_COOCH_3_ formation as R_2_ (**4**–**6**), between the temperatures *T*_max1′_ and *T*_max1a_ is observed. The emission of methane is caused by a partial cleavage of the bonds in R_2_, resulting in the emission of this gaseous decomposition product, as marked in Scheme 1.

The beginning of the emission of aromatic compounds from *T*_max1a_ for ethyl formates **1**–**3** and *T*_max1′_ for methyl acetates **4**–**6** is well seen from the gaseous FTIR spectra, shown in Figure 4. Their emission is confirmed by the appearance of the stretching vibrations of the C_Ar-H_ at 3050–3079 cm^−1^, the stretching vibrations of the C_Ar_=C_Ar_ at 1510–1580 cm^−1^, and the out-of-plane deformation vibrations of the C_Ar-H_ at 780–867 cm^−1^ [43,44,45]. The emission of aromatic fragments is visible to temperatures of 500–540 °C for all the tested compounds. However, in the gaseous FTIR spectra for compounds **1**–**3**, at temperatures above 500–540 °C, the signals coming for aromatic compounds are still visible, but they are not visible in the QMS spectra. This is caused by the emission of aromatic compounds with higher molecular masses (the formation of biphenyl compounds) which, due to their molar masses, are not detected by the QMS analyser. As it is confirmed based on the performed FTIR and QMS analyses, in Figure 4 and Figure 5, the main decomposition aromatic product for the tested molecules **1** and **4**, containing at the phenyl moiety –CH_3_, is toluene. The presence of the characteristic absorption bands at 3043–3074 cm^−1^, 2882–2935 cm^−1^, 1506–1607 cm^−1^, 694–728 cm^−1^, and the *m/z* ions 91 (C_7_H_7_^+^) and 92 (C_7_H_8_^+^) indicates the formation of this aromatic compound [46]. However, anisole is the main decomposition aromatic product in the case of compounds **2** and **5** that have the *para* –OCH_3_ group. Its formation by the occurrence of the absorption bands at 3000–3078 cm^−1^, 1502–1600 cm^−1^, 1178–1294 cm^−1^, 690–1048 cm^−1^, and the *m/z* ions 108 (C_7_H_8_O^+^) and 78 (C_6_H_6_^+^) is verified [47]. In turn, benzene is the main aromatic decomposition product for compounds **3** and **6** with –Cl as R_1_. The presence of the absorption bands at 3047–3085 cm^−1^, 1482–1500 cm^−1^, 660–1052 cm^−1^, and the *m/z* ions 77 (C_6_H_5_^+^) and 78 (C_6_H_6_^+^) that are characteristic of benzene ionisation are clearly visible from the QMS spectra collected for the tested compounds [48]. Heating the tested compounds above 500–540 °C results also in the emission of CO_2_ (the absorption bands at 669 cm^−1^ and 2300–2365 cm^−1^ and *m/z* 44) and H_2_O (the absorption bands at the range of 1100–1600 cm^−1^ and above 3500 cm^−1^ and *m/z* 18).

Taking into account the type of volatiles released, a simplified pyrolysis course of the tested compounds is presented in Scheme 1.

### 2.4. The TG/DTG Analyses (Oxidative Conditions)

**Figure 6:**
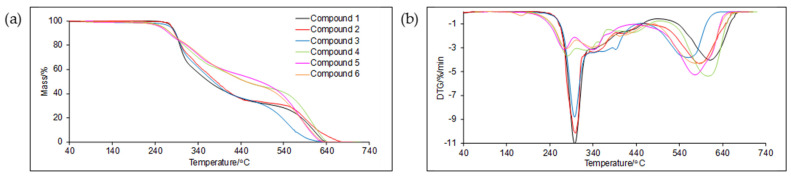
The course of the TG (**a**) and DTG (**b**) curves for the tested compounds (**1**–**6**) in an oxidative atmosphere.

Figure 6 shows the TG/DTG curves for the tested *para*-substituted heterocyclic esters **1**–**6** obtained in an oxidising atmosphere. The TG/DTG results are collected in Table 3.

As it is well visible, all the analysed compounds are characterised by high thermal stability in the presence of a synthetic air. This property may be important in the case of their approval as pharmaceuticals. Because they are stable at temperatures much higher than ambient temperature, their storage within a wide temperature range should not affect their expiration period. Previous studies on the kinetics of thermal decomposition have confirmed that highly thermally stable molecular pharmaceuticals, such as amiodarone hydrochloride or acyclovir, can be stored at temperatures ranging from 25 to 40 °C in air without shortening their shelf life [49,50]. Therefore, the high thermal stability of all nitrogen-rich heterocyclic ester derivatives (**1**–**6**) demonstrated in this study may enhance their utility as potential drug candidates and will be desirable during their processing. Additionally, these molecules may be useful for the design of highly thermally stable potential pharmaceuticals.

The decomposition of compounds with the –COOC_2_H_5_ moiety as R_2_ (**1**–**3**) begins (*T*_5%_) at temperatures of 276–280 °C. In turn, the thermal stability of compounds with the –CH_2_COOCH_3_ moiety as R_2_ (**4**–**6**) is above 250 °C. These results on the thermal stability of the tested compounds obtained in oxidative conditions are similar to those received in inert conditions. The difference in thermal stability (*T*_5%_) is only 0–4 °C for both atmospheres. This means that the type of experimental atmosphere does not affect the beginning of the decomposition of the tested compounds and thus the value of the initial decomposition energy. Ethyl formates **1**–**3** are more thermally stable compared to the methyl acetates **4**–**6**. This finding is analogous to the results obtained in an atmosphere without the addition of oxygen.

For all the analysed compounds, two clearly marked decomposition stages are seen. The first decomposition stage is composed of at least two or three steps for all the molecules tested. In the case of compounds **1**–**3**, the first step from *T*_5%_ to 330 °C with *T*_max1_ at 297–299 °C and the mass loss (Δ*m*_1_) in the range of 33.5–38.6% is observed. The second step from 330 °C to 450 °C with *T*_max1a_ and the mass loss (Δ*m*_1*a*_) in the range of 29.5–31.9% also appeared. Moreover, the second decomposition stage is spread from 450 °C to 680 °C with *T*_max2_ at 562–611 °C and the comparable mass loss (Δ*m*_2_) from 34.2% to 35.3%.

As in an inert experimental atmosphere, the first decomposition stage for compounds **4**–**6** is composed of several poorly resolved DTG signals. At this decomposition stage, one can distinguish three relatively clearly visible DTG peaks with the maxima given in Table 3. The mass loss (Δ*m*_1_) between *T*_5%_ and the temperature of 400 °C is similar for this class of compounds and is 33.5–38.8%. The mass loss in the second step (Δ*m*_1*a*_) visible from 400 °C to 460 °C is from 9.7% to 16.8%. For compounds **4**–**6** tested in the synthetic air atmosphere, the second decomposition stage with *T*_max2_ at 577–609 °C and the mass loss (Δ*m*_2_) in the range of 46.4–56.8% is clearly visible. All the tested compounds are completely decomposed under oxidising conditions because no residual mass is observed.

### 2.5. The Decomposition Course of the Tested Compounds in Oxidative Conditions

**Figure 7:**
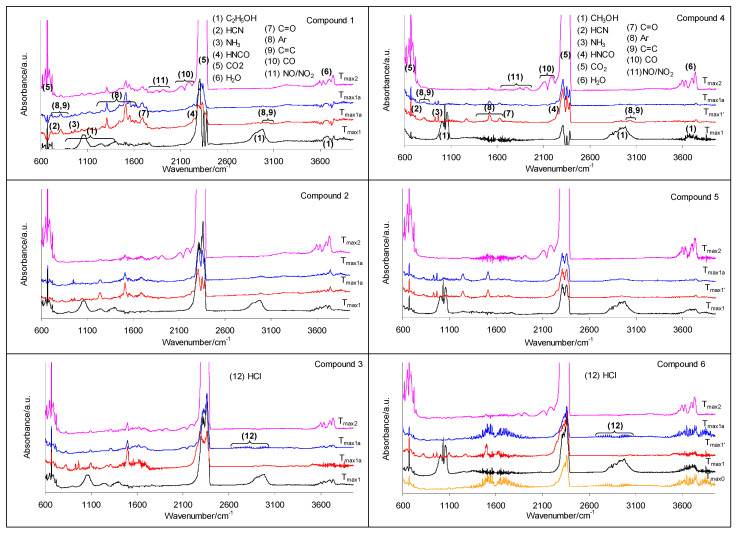
The gaseous FTIR spectra for compounds **1**–**6** collected in oxidative conditions.

**Figure 8:**
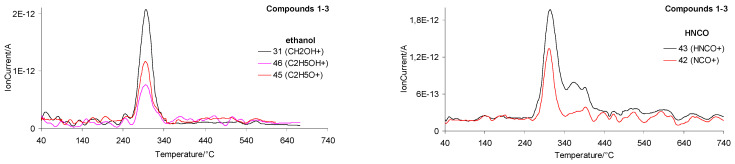

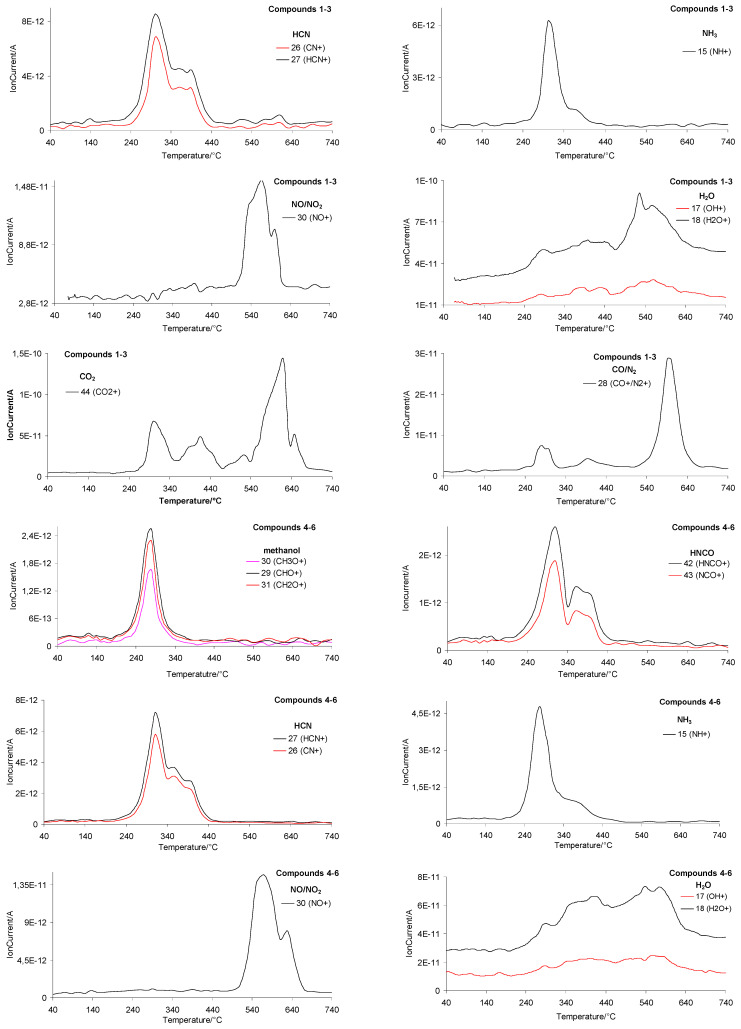

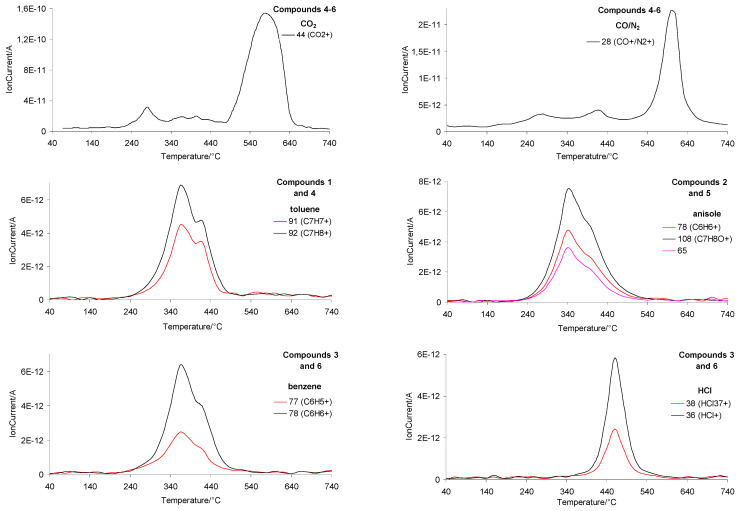
The QMS spectra for compounds **1**–**6** collected in oxidative conditions.

The gaseous FTIR spectra collected at *T*_max1_, *T*_max1′_, *T*_max1a_, and *T*_max2_ are presented in Figure 7. Additionally, the QMS spectra for the characteristic *m/z* ions of the emitted volatiles are placed in Figure 8. The gaseous FTIR spectra confirm that at *T*_max1_, the suitable alcohol (methanol or ethanol) is emitted. For ethyl formates **1**–**3**, the formation of ethanol by the appearance of the stretching vibrations for the OH (above 3600 cm^−1^), the stretching vibrations for the C–H (above 2780 cm^−1^ with the maximum at 2964 cm^−1^), the deformation vibrations for the C–H (1342–1390 cm^−1^), and the stretching vibrations for the C–O (1045–1230 cm^−1^) is clearly visible [27,28]. However, in the case of methyl acetates **4**–**6**, the emission of methanol by the presence the stretching vibrations for the C–H (2809–2954 cm^−1^), the deformation vibrations for the C–H (1330–1345 cm^−1^), and the stretching vibrations for the C–O (998–1054 cm^−1^) on the FTIR spectra is confirmed. In addition, the performed QMS analysis showed the presence of *m/z* ions formed during ionisation of these alcohols, i.e.*,* ethanol (*m/z* 31, 45, 36) and methanol (*m/z* 29, 30, 31) [29,30]. This clearly indicates the emission of these alcohols during the oxidative decomposition of the tested compounds at *T*_max1_. From the FTIR spectra collected at *T*_max1_, apart from the emission of alcohol with the appropriate structure, no emission of other compounds is observed. This may indicate, as in the case of an inert atmosphere, that the emission of other gases by the absorption signals of alcohol is obscured. The collected QMS spectra prove that the volatile emission at *T*_max1_ is more diverse. Just like in an inert atmosphere, in this temperature range, HCN (FTIR: the absorption signal at 713 cm^−1^; *m/z*: 27 (HCN^+^) and 26 (CN^+^)), HNCO (FTIR: peaks at 2270–2290 cm^−1^, *m/z* ions: 43 (HNCO^+^), 42 (NCO^+^)), NH_3_ (FTIR: two bands at 931 cm^−1^ and 966 cm^−1^; *m/z*: 15 (NH^+^)), and some amounts of CO (FTIR: the peaks at 2088–2167 cm^−1^; *m/z*: 28 CO^+^), CO_2_ (FTIR: the peaks at 2300–2365 cm^−1^ and 669 cm^−1^; *m/z*: 44 CO_2_^+^), H_2_O (FTIR: the signals at 3450–4000 cm^−1^; *m/z*: 18 H_2_O^+^), 17 (OH^+^)), and N_2_ (*m/z*: 28 (N_2_^+^) are also formed [51,52].

Comparing the emission intensity of gaseous organic compounds in an inert atmosphere and in an oxidising atmosphere, it can be concluded that it is lower in the air atmosphere. This may indicate that gaseous organic compounds combustion reactions partially occur in the presence of oxygen.

At *T*_max1a_ (compounds **1**–**3**) and *T*_max1′_ (compounds **4**–**6**) the continuous emission of HCN, HNCO, NH_3_, and N_2_ based on the FTIR and QMS is confirmed. The emission of these organic gases stops at ca. 440 °C. Additionally, at *T*_max1a_ (compounds **1**–**3**) and *T*_max1′_ (compounds **4**–**6**), the emission of aromatic and carbonyl compounds begins, as evidenced by the presence of the following FTIR absorption bands: 1675–1681 cm^−1^ (the stretching vibrations for the C=O characteristic for carbonyl compounds) and 780–850 cm^−1^ (the out-of-plane deformation vibrations of the C_Ar-H_), 1510–1602 cm^−1^ (the stretching vibrations of the C_Ar_=C_Ar_), and 3050–3060 cm^−1^ (the stretching vibrations of the C_Ar-H_).

As the heating temperature increases above 440 °C, the creation of CO, CO_2_, H_2_O, and especially the formation of nitrogen oxides (FTIR: 1858–1950 cm^−1^ (the stretching vibrations of the NO in nitrogen monoxide) and 1564–1633 cm^−1^ (the stretching vibrations of the NO in nitrogen dioxide_,_ QMS: *m/z* ion 30 NO^+^)) and N_2_ (*m/z*: 28) confirms the earlier supposition that some of the emitted volatiles undergo oxidation reactions. As it is well visible from the gaseous FTIR and QMS spectra, the emission of nitrogen oxides starts at lower temperatures (ca. 340 °C). However, at this temperature, their emission is low. Their high emission at temperatures above 500 °C is observed. This confirms that oxidation reactions of gaseous decomposition products containing nitrogen in their structure occur at a higher rate at higher temperatures. This also proves that at lower temperatures, intermediate products resulting from the reaction between radicals and oxygen are formed.

In addition, no methane emission in the case of the decomposition process of compounds **4**–**6** is observed. This confirms that methane under this analysis conditions is not formed or that it is burned to CO_2_ and H_2_O. Moreover, the creation of HCl for compounds **3** and **6**—containing –Cl at the phenyl moiety—at *T*_max1a_ as characteristic “noises” in the range of 2100–3100 cm^−1^ is confirmed [40,41].

**Figure 9:**
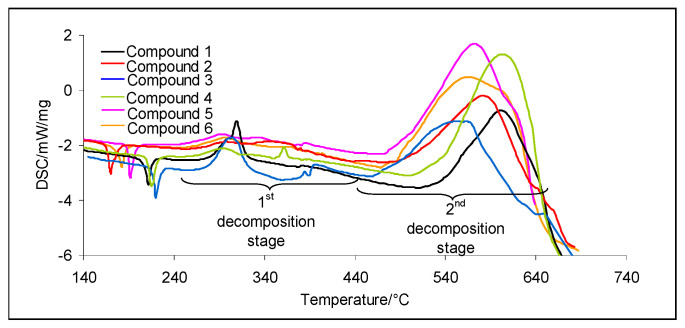
The course of the DSC curves of compounds **1**–**6** in an air atmosphere.

**Scheme 2:**
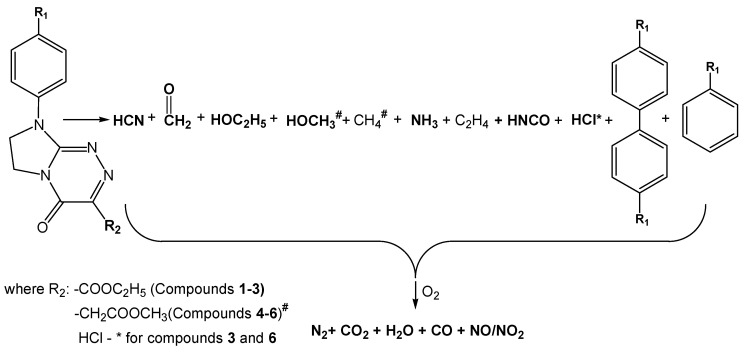
The emitted volatile compounds in an oxidative atmosphere.

In order to prove the oxidation reactions in the gaseous phase of emitted volatiles and/or formed residues, the course of the DSC curves collected in an air atmosphere up to 740 °C is presented in Figure 9. These DSC curves show the presence of mainly exothermic signals connected with the decomposition process of the tested compounds. The first exothermic DSC signal between 240 °C and 440 °C is observed. This exothermic DSC signal of temperature range covers with the first decomposition stage temperature range appointed from the TG/DTG analysis. These results confirm that the chemical reactions of the volatiles/residues with oxygen already occur at this temperature range. The second exothermic DSC signal with high intensity above 440 °C is observed. This proves the previous assumptions that volatiles oxidation/combustion reactions occur more efficiently at higher temperatures, as shown in Scheme 2.

### 2.6. Haemolytic Activity of the Investigated Heterocyclic Esters (**1**–**6**)

The effect of active newly synthesised compounds on living cells, such as blood cells, is a key factor in drug design and development. At this stage of research, it is necessary to carry out a preliminary screening for the toxicity profile of each potential drug. Various tests can be used for this purpose. An alternative tool for evaluating drug candidates’ toxicity is to perform a haemolysis assay on red blood cells. Mammalian erythrocytes, the most numerous cells in the blood, seem to be an excellent model for studying this cytotoxicity. They are widely used, due to not only their easy availability, but also the similarity of the erythrocyte membrane to other cell membranes. The haemolytic activity of potential drug candidates is an indicator of overall cytotoxicity to normal cells. Therefore, measuring haemolysis is an important tool in many fields, including the development of potential drugs. Additionally, testing the haemolytic potential (haemocompatibility) is necessary for active pharmaceutical ingredients and their carriers intended for intravenous injection because it provides a direct indication of the toxicity of these injectable preparations [53,54,55,56,57]. We applied a haemolysis test to evaluate the toxicity of potential ester drugs (**1**–**6**) to important blood components, i.e., red blood cells. The haemolytic activity of the tested compounds was compared with that of Triton X-100 in the same erythrocyte model. As shown in Table 4, Triton induced complete haemolysis, while all the tested compounds **1**–**6** were not toxic to red blood cells as they did not promote any haemolytic effects at a concentration at which they were pharmacologically active. The results of this study confirmed a good haemocompatibility of the tested esters (a haemolytic activity lower than 3.5%), which would make them suitable candidates for further in vivo investigations.

### 2.7. Protective Effect of the Investigated Heterocyclic Esters (**1**–**6**) against Haemolysis Induced by Oxidative Stress

The ability of the investigated heterocyclic esters (**1**–**6**) to counteract oxidative stress-induced haemolysis was studied in a model of erythrocytes exposed to AAPH-derived peroxyl radicals or hydrogen peroxides. These oxidative agents induce protein oxidation and lipid peroxidation, leading to membrane damage of red blood cells and ultimately, to haemolysis. Some compounds may prevent oxidation damage to membrane proteins and lipids and thus inhibit haemolysis. As shown in Table 4, the inhibition of oxidative haemolysis was dependent on the type of oxidant (peroxyl radicals or hydrogen peroxides) and the structure of the tested compound. The investigated molecules were better at preventing oxidative damage to erythrocytes caused by peroxyl radicals than by hydrogen peroxides. Among heterocyclic ethyl formates (**1**–**3**), the most active in inhibiting AAPH-generated oxidative haemolysis proved to be compounds **1** and **3**, bearing the methyl and chloro group at the phenyl ring, respectively. In addition, the compound **2** (with the methoxy substituent) was almost equally effective in counteracting the haemolysis caused by these radicals. In turn, among heterocyclic methyl acetates (**4**–**6**), the compound **6** (with the chloro group) was the most effective in protecting red blood cells from AAPH-induced damage. The most active in inhibiting the haemolysis induced by H_2_O_2_ turned out to be two structures—both with methyl substitution—**1** (the ethyl formate derivative) and **4** (the methyl acetate derivative). These results show that the above-mentioned esters may protect cell membranes from oxidative stress-induced damage as they inhibit haemolysis in erythrocytes exposed to oxidative agents.

Both the lack of haemolytic activity (which is an indicator of general cytotoxicity to normal cells) and the protection of red blood cells from oxidative damage are beneficial properties of the heterocyclic esters evaluated in the preclinical phase of drug development, which may increase the potential therapeutic utility of these drug candidates. Additionally, these molecules can serve as lead structures for the design of agents to prevent oxidative stress-mediated diseases.

## 3. Materials and Methods

### 3.1. Heterocyclic Esters (**1**–**6**)

Ethyl 4-oxo-8-[4-(R-phenyl)]-4,6,7,8-tetrahydroimidazo[2,1-*c*][1,2,4]triazine-3-carboxylates (**1**–**3**) were synthesised from 2-hydrazinylidene-1-[4-(R-phenyl)]imidazolidine (where R denotes –CH_3_ or –CH_3_O or –Cl) and diethyl 2-(hydroxyimino)malonate, using a previously patented and published synthesis method [1,2]. In turn, methyl {4-oxo-8-[4-(R-phenyl)]-4,6,7,8-tetrahydroimidazo[2,1-*c*][1,2,4]triazin-3-yl}acetates (**4**–**6**) were prepared from 2-hydrazinylidene-1-[4-(R-phenyl)]imidazolidine (where R denotes –CH_3_ or –CH_3_O or –Cl) and dimethyl but-2-ynedioate, as earlier reported [5]. The correct structures of these heterocyclic esters **1**–**6** have previously been established using spectroscopic data (^1^H NMR, IR and EI-MS spectra) and elemental analyses within ±0.4% of the theoretical values for C, H, N [1,2,5].

### 3.2. Differential Scanning Calorimetry (DSC)

The results from the DSC made it possible to determine the melting temperatures of the tested compounds including the onset melting temperatures (*T*_onset_) and the maximum melting temperatures (*T*_melt_) as well as the melting enthalpy (Δ*H*). The DSC analyses with a use of a DSC 204 Phoenix apparatus (Netzsch, Selb, Germany) were applied. The tested compounds (a sample mass of approx. 10 mg) in the aluminium crucible with a pierced lid were heated from 20 °C to 250 °C. The heating rate was 10 °C min^−1^. The course of melting was studied in inert and oxidative atmospheres. For an inert atmosphere, helium with a flow rate of 40 mL min^−1^ was used. For an oxidative atmosphere, a synthetic air with a flow rate of 40 mL min^−1^ was applied.

### 3.3. Thermogravimetric Analysis/Differential Scanning Calorimetry Coupled On-Line with FTIR and QMS Analysers (TG/DTG/DSC/FTIR/QMS)

The TG/DTG/DSC analyses were performed with the use of a STA 449 Jupiter F1 instrument produced by Netzsch, Selb, Germany. All the compounds (a sample mass of approx. 10 mg) in the solid form in open corundum crucibles were placed and heated from 40 °C to 900 °C. The applied heating rate was 10 °C min^−1^. The analyses in an inert atmosphere (helium with a flow rate of 40 mL min^−1^) and in an oxidative atmosphere (a synthetic air with a flow rate of 100 mL min^−1^) were carried out. The results obtained from the TG/DTG curves allowed for the determination of the following values: the initial decomposition temperature marked as a 5% of mass loss (*T*_5%_), peak maximum decomposition temperatures (*T*_max_), mass losses in each decomposition stage (Δ*m*), and the residual mass after heating to 900 °C (rm). In turn, the additional DSC curves made it possible to evaluate the exothermic/endothermic changes that occurred in the samples during heating.

In addition, a STA 449 Jupiter F1 instrument was connected on-line with two gas analysers, namely a Fourier Transform Infrared Spectrometer (a FTIR TGA 585, Bruker, Mannheim, Germany) and a Quadrupole Mass Spectrometer (a QMS 403 C Aëolos, Netzsch, Selb, Germany). This connection of the TG/DTG/DSC apparatus with gas analysers allowed for the qualitative identification of volatile products released during the decomposition of the tested compounds. Thus, the decomposition path of the tested compounds was proposed. In the case of the FTIR analyser, the gaseous FTIR spectra within the wavenumber range of 600–4000 cm^−1^, with a resolution of 4 cm^−1^, and 32 scans per spectrum were collected. In turn, the gaseous QMS spectra between 10 and 150 amu were gathered. Both analysers were equipped with the heated cell and transfer line in order to minimise the effect of condensation of volatiles. An IR cell and a Teflon transfer line were heated to 200 °C. A QMS inlet system was heated to 200 °C and the quartz capillary line to 300 °C.

### 3.4. Haemolytic Activity Assay

The ability of the investigated heterocyclic esters **1**–**6** to induce haemolysis was assessed ex vivo in a mammalian red blood cell model. The blood was collected from two rats (male Wistar rats; 8 weeks old; weighing 240–250 g; the Experimental Medicine Centre, Medical University of Lublin, Poland) into tubes containing heparin (Li-Heparin: 16 IU mL^−1^) as an anticoagulant. The tubes were centrifuged at 1000 rpm for 10 min at 4 °C, the supernatant and buffy coat were carefully removed, and the erythrocytes were resuspended with phosphate-buffered saline (PBS; pH 7.4; Biomed, Lublin, Poland) and centrifuged as previously indicated. The washing procedure was repeated until a transparent supernatant was obtained. The washed red blood cells were finally resuspended in PBS to a final concentration of 4%. Such a suspension was used for further studies. Each compound at a concentration of 0.15 mM was added to the erythrocyte suspension. Red blood cells in PBS and in 1% Triton X-100 solution (Sigma-Aldrich, Saint Louis, MO, USA) were considered negative (0% lysis) and positive (100% lysis) controls, respectively. The reaction mixtures were gently shaken in a water bath at 37 °C for 60 min. Then, the samples were centrifuged at 1500 rpm for 10 min and the absorbance of the supernatants was measured at λ_max_ = 540 nm on a Hitachi U2800 spectrophotometer (Hitachi, Tokyo, Japan). The experiment was repeated three times, and the results are given as the average of three replicates.

### 3.5. Oxidative Haemolysis Inhibition Assay

The ability of the investigated heterocyclic esters **1**–**6** to counteract haemolysis induced by oxidative stress was evaluated ex vivo in a mammalian erythrocyte model. For this purpose, each compound at a concentration of 0.15 mM was incubated for 60 min in a water bath at 37 °C with a suspension of red blood cells prepared as described in the haemolysis assay. Freshly prepared ice-cold solutions of 40 mM 2,2′-azobis(2-amidinopropane)dihydrochloride (AAPH; Sigma-Aldrich, Saint Louis, MO, USA) or 75 mM hydrogen peroxide (H_2_O_2_; Sigma-Aldrich, Saint Louis, MO, USA) in PBS were then added to each sample. The reaction mixtures were gently shaken in a water bath at 37 °C for 210 min (samples with AAPH) or 180 min (samples with H_2_O_2_). After incubation, the samples were centrifuged at 1500 rpm for 5 min and the absorbance of the supernatants containing AAPH or H_2_O_2_ was measured spectrophotometrically at λ_max_ = 524 or 540 nm, respectively. The experiment was repeated three times, and the results are given as the average of three replicates. The antihaemolytic activity of the tested compounds was calculated in relation to antioxidant standards, i.e., ascorbic acid or Trolox.

## 4. Conclusions

This paper presented the thermal properties and decomposition course of two types of *para*-R_1_-substituted nitrogen-rich heterocyclic esters with prospective pharmaceutical utility. The detailed thermal characterisation of these small molecules was carried out during the preclinical phase of drug development. The DSC analysis proved high purity of all the tested compounds and showed that they melted above 170 °C in both atmospheres (inert and oxidising). Among all the studied esters, those with a methyl group at the *para* position of the phenyl moiety exhibited the highest melting temperature. TG/DTG analyses confirmed the high thermal stability (above 250 °C) of all the tested compounds in helium and air. Ethyl formates **1**–**3** were characterised by ca. 13–23 °C higher thermal stability compared to methyl acetates **4**–**6** in both atmospheres. The decomposition process of the tested molecules was multi-stage, including at least two main stages in which two/three thermal steps were distinguished. The simultaneous TG/FTIR/QMS analyses proved that the pyrolysis and the oxidative decomposition were complicated processes and occurred as simultaneous decomposition reactions of various bonds with the same or similar decomposition activation energies. In inert conditions, mainly the homolytic decomposition of the C–C, C–N, and C–O bonds was confirmed, leading to the formation of radicals based on the type of the volatiles formed. As a result, the emission of alcohol (ethanol (in the case of ethyl formates **1**–**3**) or methanol (in the case of methyl acetates **4**–**6**)), NH_3_, HNCO, HCN, aldehydes, ethane, CO_2_, H_2_O, CH_4_, HCl and aromatics was confirmed. In turn, under oxidative conditions, the concentration of emitted volatiles, namely ethanol (in the case of ethyl formates **1**–**3**) or methanol (in the case of methyl acetates **4**–**6**), NH_3_, HNCO, HCN, HCl, aldehydes, ethane, and aromatic compounds, was lower as compared to inert conditions. In the case of compounds **4**–**6**, no emission of CH_4_ was observed. In addition, the formation of new gaseous products, such as NO/NO_2_, CO, and N_2_, as a result of some oxidation and combustion processes, was confirmed. This proved that the decomposition course of the studied compounds in air was more complicated than in an oxygen-free atmosphere. The obtained results proved the high thermal stability, purity, and no polymorphic transformations (when screened for polymorphic behaviour at a low heating rate) of the tested compounds. Therefore, the performed studies are an important contribution to the current knowledge and will be of practical significance when establishing the storage and processing conditions of the investigated molecules, as well as their impact on the environment in the event of thermal utilisation. The beneficial thermal properties of the studied compounds may increase interest in these potential drug candidates. The lack of polymorphic transformations in the solid state of the investigated heterocyclic esters, as well as their high thermostability and purity seem to predispose them to future pharmaceutical applications. Additionally, all the tested nitrogen-rich heterocyclic esters proved to be safe for red blood cells, and some of them protected erythrocytes from AAPH- and H_2_O_2_-induced stress, which is an important feature of potential drug candidates. This research resulted in the documentation of beneficial thermal behaviour and biological properties of a number of original structures that represent an excellent material for the future development and implementation of these potential drugs.

## Figures and Tables

**Table 1:** The DSC results for the tested compounds (**1**–**6**).

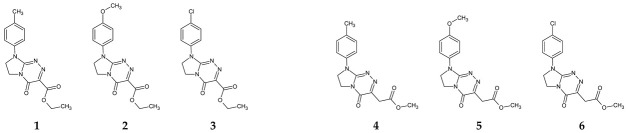						
Compound	Inert Atmosphere			Oxidative Atmosphere		
	*T*_onset_/°C	*T*_melt_/°C	Δ*H*/J/g	*T*_onset_/°C	*T*_melt_/°C	Δ*H*/J/g
**1**	213	215	135.8	212	215	147.3
**2**	171	173	152.2	170	172	161.6
**3**	207	210	143.6	206	211	151.9
**4**	218	220	123.8	217	221	134.3
**5**	194	196	135.1	194	196	143.9
**6**	183	185	126.7	182	185	135.1

**Table 2:** TG/DTG data for the tested compounds (**1**–**6**) (inert conditions).

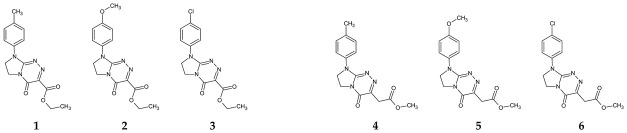								
Compound	Decomposition Process							
	First Decomposition Stage					Second Decomposition Stage		
	*T*_5%_/°C	*T*_max1_/°C	Δ*m*_1_/%	*T*_max1a_/°C	Δ*m*_1*a*_/%	*T*_max2_/°C	Δ*m*_2_/%	rm/%
**1**	278	301	40.0	354/448	32.0	726	28.0	0
**2**	273	298	36.9	345/498	31.8	715	31.3	0
**3**	280	300	38.7	382	37.1	733	24.2	0
	***T*_5%_/°C**	***T*_max1_/*T*_max1′_°C**	**-**	***T*_max1a_/ °C**	**Δ*m*_1_ *_+_* Δ*m*_1*a*_/%**	***T*_max2_/°C**	**Δ*m*_2_/%**	**rm/%**
**4**	260	288/309	-	407	65.7	-	9.0	25.3
**5**	256	278/326	-	486	68.4	-	4.0	27.6
**6**	257	280/335	-	381/401	63.1	-	9.3	27.5

**Table 3:** TG/DTG data collected for the tested compounds (**1**–**6**) (oxidising conditions).

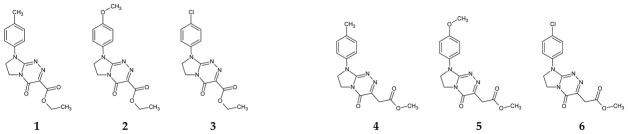							
Compound	Decomposition Process						
	First Decomposition Stage					Second Decomposition Stage	
	*T*_5%_/°C	*T*_max1_/*T*_max1′/_°C	Δ*m*_1_/%	*T*_max1a_/°C	Δ*m*_1*a*_/%	*T*_max2_/°C	Δ*m*_2_/%
**1**	280	297	38.6	359/431	29.5	611	35.2
**2**	277	299	33.9	357/422	31.9	595	34.2
**3**	276	297	33.5	364/393	31.2	562	35.3
**4**	259	281/326	36.8	433	16.8	609	46.4
**5**	252	274/339	33.5	383	9.7	577	56.8
**6**	257	279/337	38.8	403	13.8	581	47.4

**Table 4:** Haemolytic and antihaemolytic activities of the investigated heterocyclic esters (**1**–**6**).

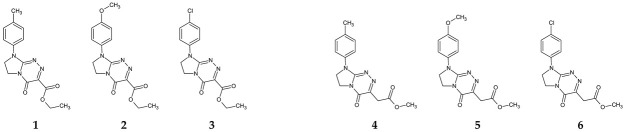			
Compound/Control	Haemolytic Activity (%) ^a^	Inhibition (%) of Oxidative Haemolysis	
		Induced by AAPH ^b^	Induced by H_2_O_2_ ^c^
**1**	3.08 ± 0.22	81 ± 6.7	75 ± 7.0
**2**	3.29 ± 0.24	77 ± 5.5	48 ± 3.3
**3**	2.64 ± 0.11	81 ± 5.8	40 ± 3.8
**4**	3.13 ± 0.19	64 ± 4.0	82 ± 7.1
**5**	3.46 ± 0.25	61 ± 4.8	53 ± 3.8
**6**	3.05 ± 0.24	79 ± 6.1	52 ± 4.1
Triton X-100	100	-	-
Ascorbic acid	-	100	-
Trolox	-	-	100

AAPH—2,2′-azobis(2-methylpropionamidine)dihydrochloride; H_2_O_2_—hydrogen peroxide; Trolox—6-hydroxy-2,5,7,8-tetramethylchroman-2-carboxylic acid; ^a^—haemolytic activity of compounds **1**–**6** in relation to a positive control, i.e., 1% Triton X-100 solution; ^b^—antihaemolytic activity (in the model of rat erythrocytes exposed to AAPH) of compounds **1**–**6** in relation to ascorbic acid; ^c^—antihaemolytic activity (in the model of rat erythrocytes exposed to H_2_O_2_) of compounds **1**–**6** in relation to Trolox. Compounds were tested at a concentration of 0.15 mM. Data (from three independent experiments) are shown as the mean ± standard deviation.

## Data Availability

The data presented in this study are available on request from the authors.
